# Supplementary material for: Language barriers in global bird conservation
Source: PLoS One. 2022 Apr 20;17(4):e0267151. doi: 10.1371/journal.pone.0267151 (PMC9020734; doi:10.1371/journal.pone.0267151)
Supplement: S2 Table — The order of each species was also incorporated in the models as a random factor. Statistically significant p-values (p < 0.05) are indicated in bold. (DOCX) [file pone.0267151.s002.docx]

|  |  |  |  |  |  |  |  |  |  |  |
| --- | --- | --- | --- | --- | --- | --- | --- | --- | --- | --- |
|  |  | **Official languages** | | | |  | **Most Spoken languages** | | | |
| Predictor |  | **Estimate** | **Std. error** | **Z-value** | **p** |  | **Estimate** | **Std. error** | **Z-value** | **p** |
| Intercept |  | -2.26 | 0.07 | -30.49 | ***<0.001*** |  | -3.42 | 0.09 | -37.37 | ***<0.001*** |
| *Coefficients* |  |  |  |  |  |  |  |  |  |  |
| Log10(Area) |  | 0.67 | 0.01 | 78.15 | ***<0.001*** |  | 0.82 | 0.01 | 82.3 | ***<0.001*** |
| Migrant |  | 0.31 | 0.02 | 15.4 | ***<0.001*** |  | 0.22 | 0.02 | 9.92 | ***<0.001*** |
| Threat category (CR) |  | 0.32 | 0.07 | 4.35 | ***<0.001*** |  | 0.41 | 0.09 | 4.82 | ***<0.001*** |
| Threat category (DD) |  | 0.28 | 0.13 | 2.24 | **0.03** |  | 0.22 | 0.15 | 1.52 | 0.13 |
| Threat category (EN) |  | 0.12 | 0.05 | 2.61 | ***0.01*** |  | 0.17 | 0.05 | 3.06 | ***0.002*** |
| Threat category (NT) |  | 0.02 | 0.03 | 0.84 | 0.4 |  | 0.01 | 0.03 | 0.19 | 0.85 |
| Threat category (VU) |  | 0.03 | 0.03 | 0.82 | *0.41* |  | 0.01 | 0.04 | 0.13 | *0.89* |
|  |  |  |  |  |  |  |  |  |  |  |

**S2 Table.** Results of negative binomial generalized linear mixed models (GLMM) to explain the number of (official or most spoken) languages spoken within bird species distribution (the response variable) using the three explanatory variables: log_10_-transformed distribution range size (km^2^), migratory status (non-migrant as the reference category), and IUCN threat categories (Least Concern as the reference category). The order of each species was also incorporated in the models as a random factor. Statistically significant p-values (p < 0.05) are indicated in bold.
