# Supplementary material for: Language barriers in global bird conservation
Source: PLoS One. 2022 Apr 20;17(4):e0267151. doi: 10.1371/journal.pone.0267151 (PMC9020734; doi:10.1371/journal.pone.0267151)
Supplement: S1 Fig — (DOCX) [file pone.0267151.s005.docx]

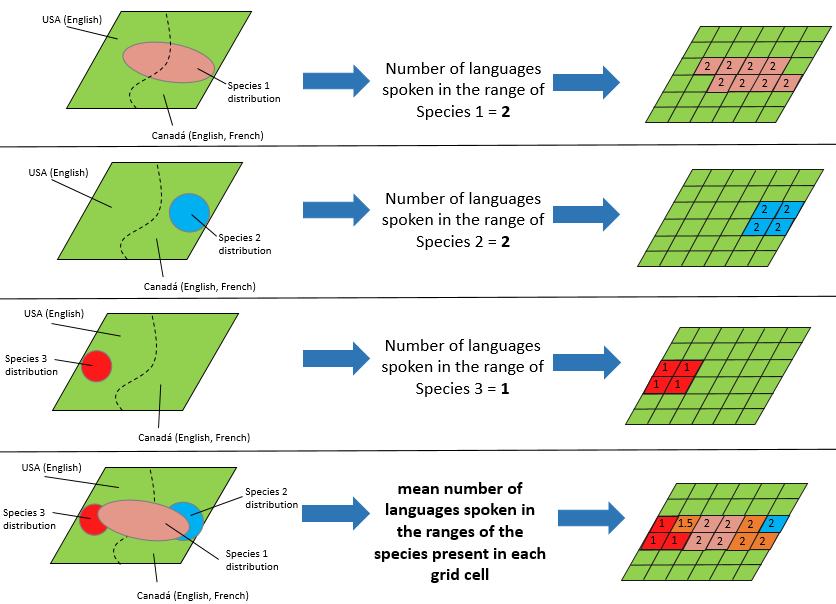


**S1 Figure.** Methodological framework for mapping mean linguistic diversity across all species within each grid cell
