# Supplementary material for: Language barriers in global bird conservation
Source: PLoS One. 2022 Apr 20;17(4):e0267151. doi: 10.1371/journal.pone.0267151 (PMC9020734; doi:10.1371/journal.pone.0267151)
Supplement: S2 Fig — (a) Number of most spoken languages within the distributions of all bird species (n = 10,863), threatened species (n = 1427) and migratory species (n = 1939). (b) Number of most spoken languages spoken in the distributions of bird species by threat category (as assessed by the International Union for Conservation of Nature). (DOCX) [file pone.0267151.s006.docx]

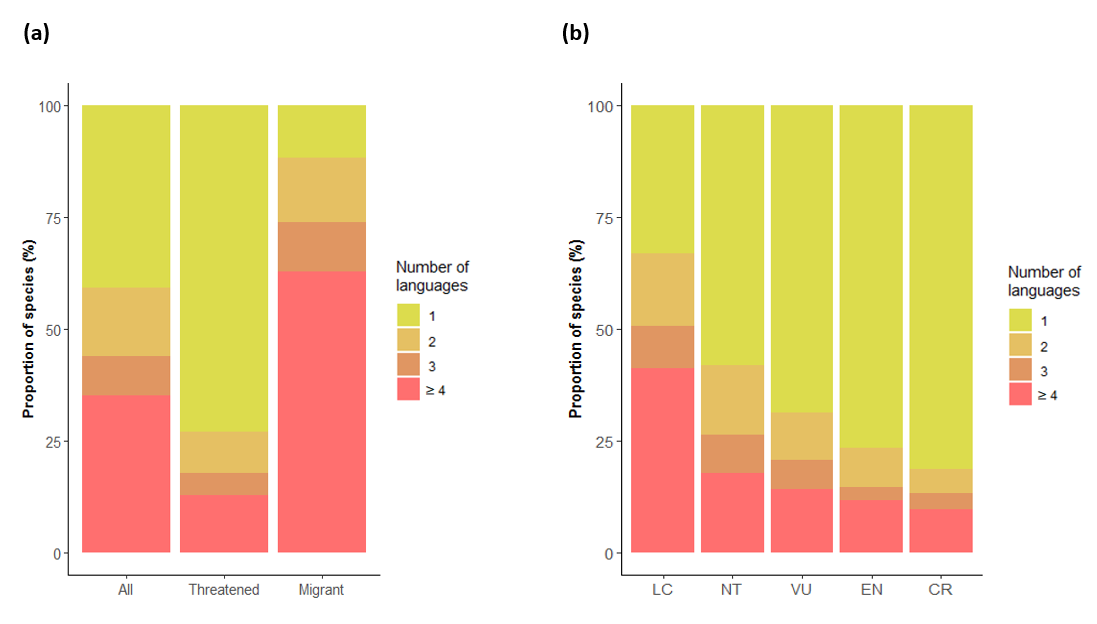


**S2 Figure.** Language diversity among bird distributions. **(a)** Number of most spoken languages within the distributions of all bird species (n=10,863), threatened species (n=1427) and migratory species (n=1939). **(b)** Number of most spoken languages spoken in the distributions of bird species by threat category (as assessed by the International Union for Conservation of Nature).
