# Supplementary material for: Language barriers in global bird conservation
Source: PLoS One. 2022 Apr 20;17(4):e0267151. doi: 10.1371/journal.pone.0267151 (PMC9020734; doi:10.1371/journal.pone.0267151)
Supplement: S3 Fig — (a) Relationship between bird species’ distribution range size and the number of languages within their distribution. International Union for Conservation of Nature (IUCN) threat categories are shown in different colours. Number of languages spoken within each species’ distribution by (b) migratory status and IUCN threat categories, and by (c) taxonomic order. This analysis was done using the dataset of most spoken language in each country. (DOCX) [file pone.0267151.s007.docx]

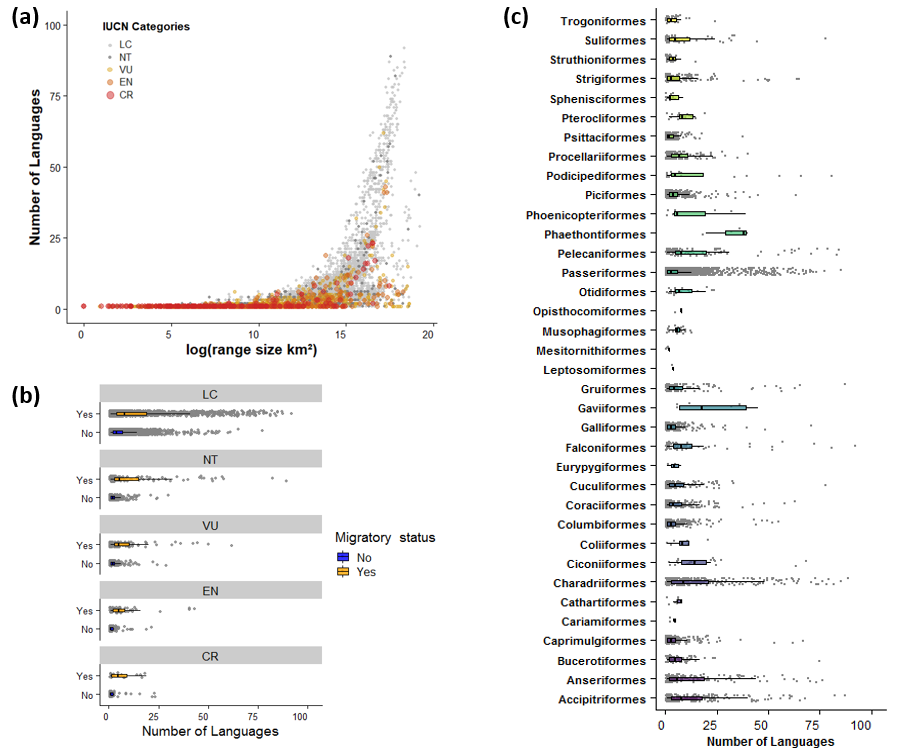


**S3 Figure. (a)** Relationship between bird species’ distribution range size and the number of languages within their distribution. International Union for Conservation of Nature (IUCN) threat categories are shown in different colours. Number of languages spoken within each species’ distribution by **(b)** migratory status and IUCN threat categories, and by **(c)** taxonomic order. This analysis was done using the dataset of most spoken language in each country.
