# Supplementary material for: Language barriers in global bird conservation
Source: PLoS One. 2022 Apr 20;17(4):e0267151. doi: 10.1371/journal.pone.0267151 (PMC9020734; doi:10.1371/journal.pone.0267151)
Supplement: S4 Fig — Number of official languages associated with (a) all species (n = 10,863), (b) migratory species (n = 1,939), and (c) threatened species (n = 1,427). The same analysis but for most spoken languages in each country for (d) all species, (e) migratory species, and (f) threatened species. The number of species associated only with the language is shown in orange and the number of species associated with the language and one or more other languages is shown in blue. (DOCX) [file pone.0267151.s008.docx]

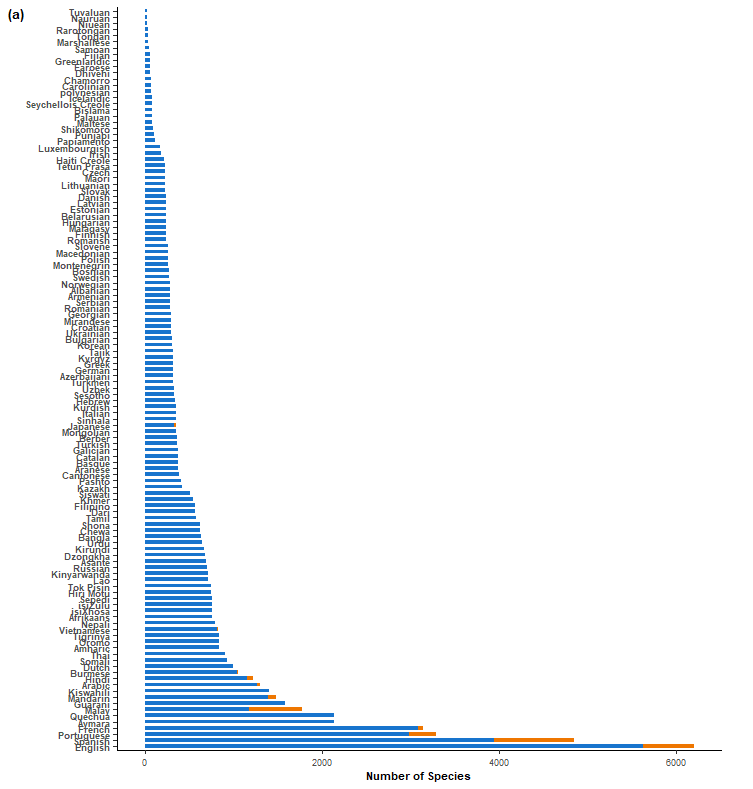


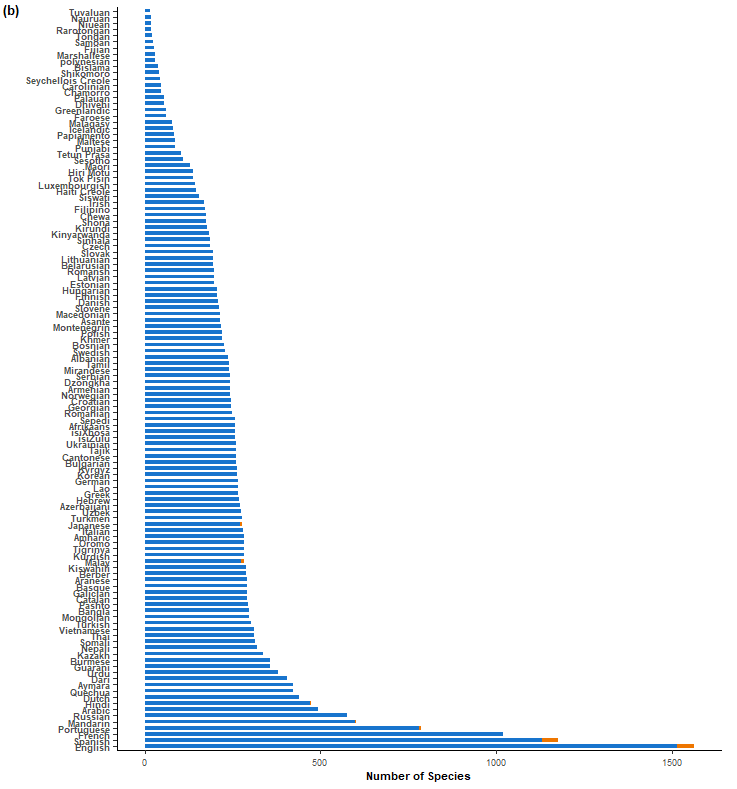


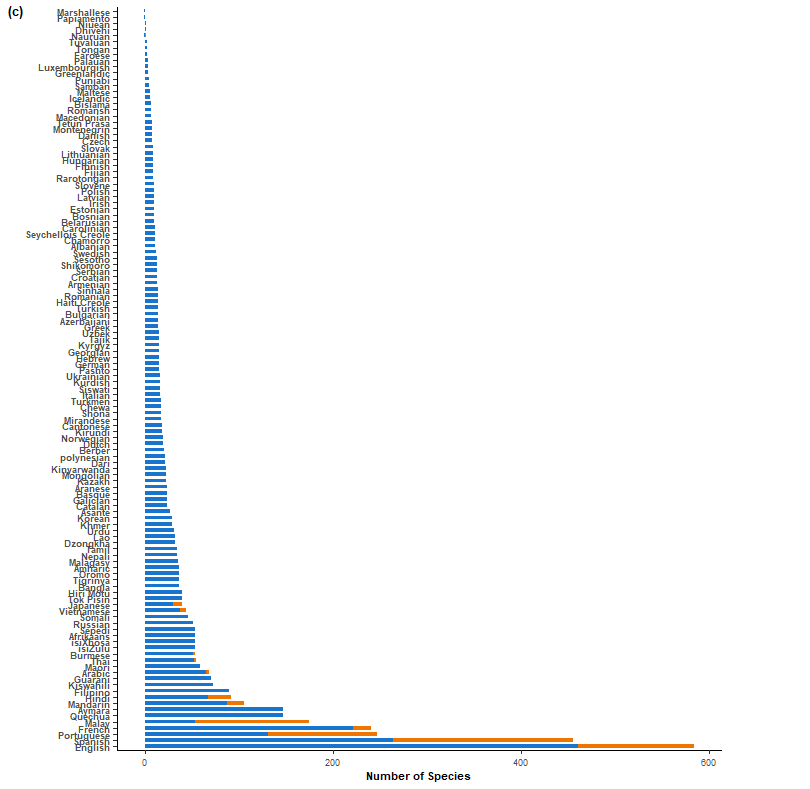


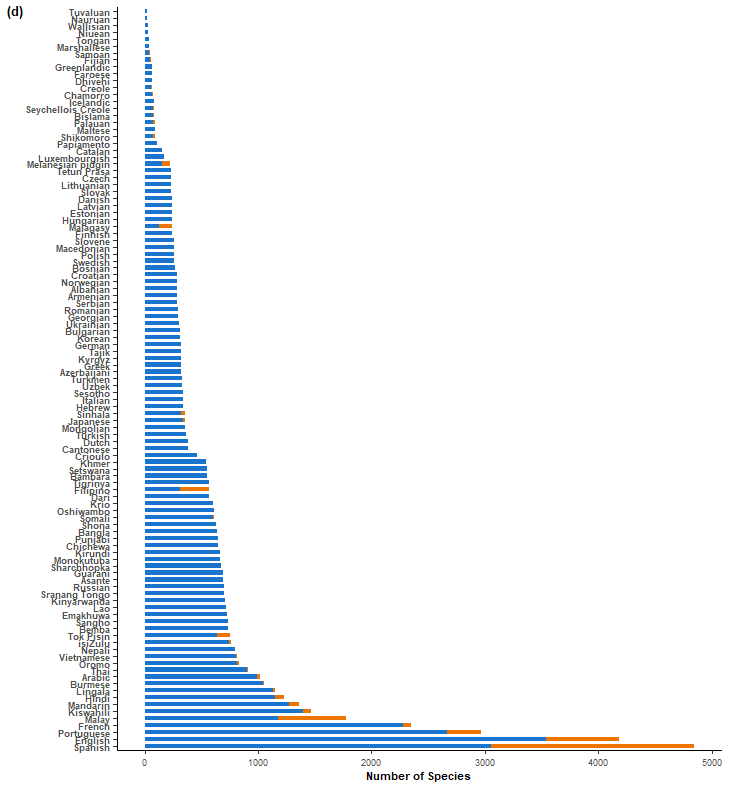


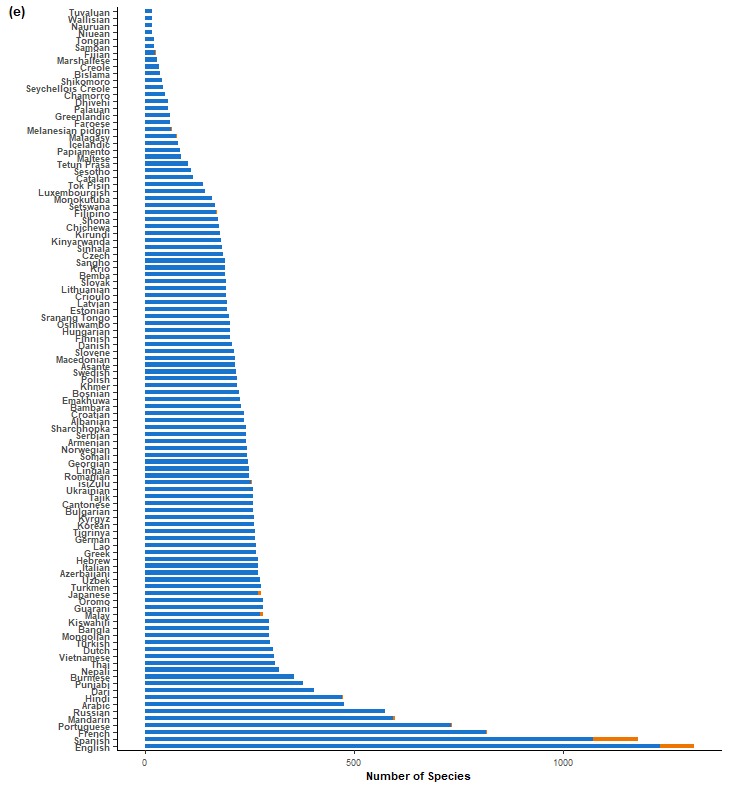


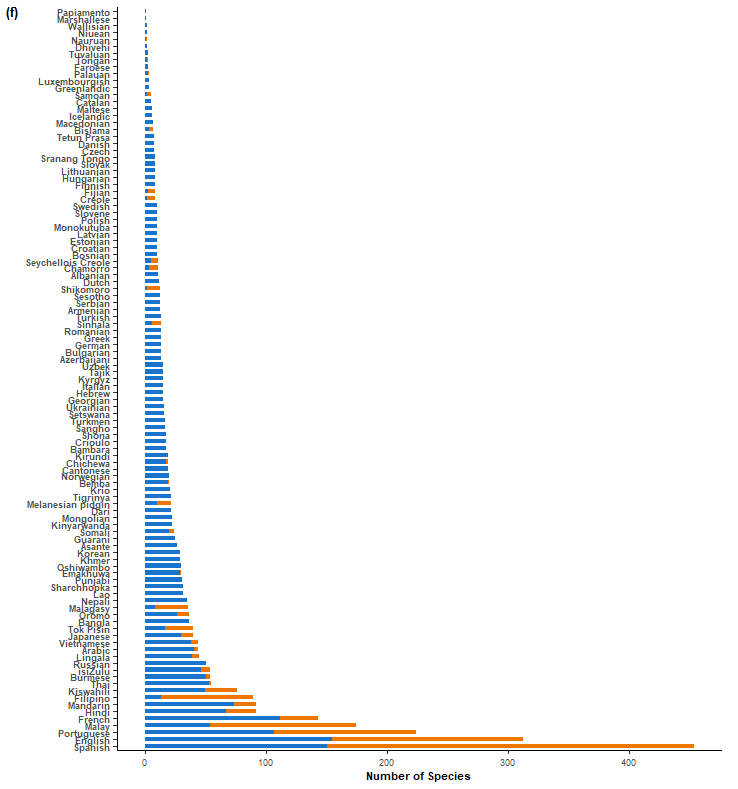


**S4 Figure.** Number of bird species associated with a particular language. Number of official languages associated with **(a)** all species (n=10,863), **(b)** migratory species (n=1,939), and **(c)** threatened species (n=1,427). The same analysis but for most spoken languages in each country for **(d)** all species, **(e)** migratory species, and **(f)** threatened species. The number of species associated only with the language is shown in orange and the number of species associated with the language and one or more other languages is shown in blue.
