# Supplementary material for: Language barriers in global bird conservation
Source: PLoS One. 2022 Apr 20;17(4):e0267151. doi: 10.1371/journal.pone.0267151 (PMC9020734; doi:10.1371/journal.pone.0267151)
Supplement: S5 Fig — Bivariate maps showing the number of species (species richness) and the mean number of languages within the distribution of species found within each 30km × 30km grid cell for (a) all bird species, (b) threatened bird species, and (c) migratory bird species. The number of languages within each species’ distribution was calculated using the most spoken languages in each country. (DOCX) [file pone.0267151.s009.docx]

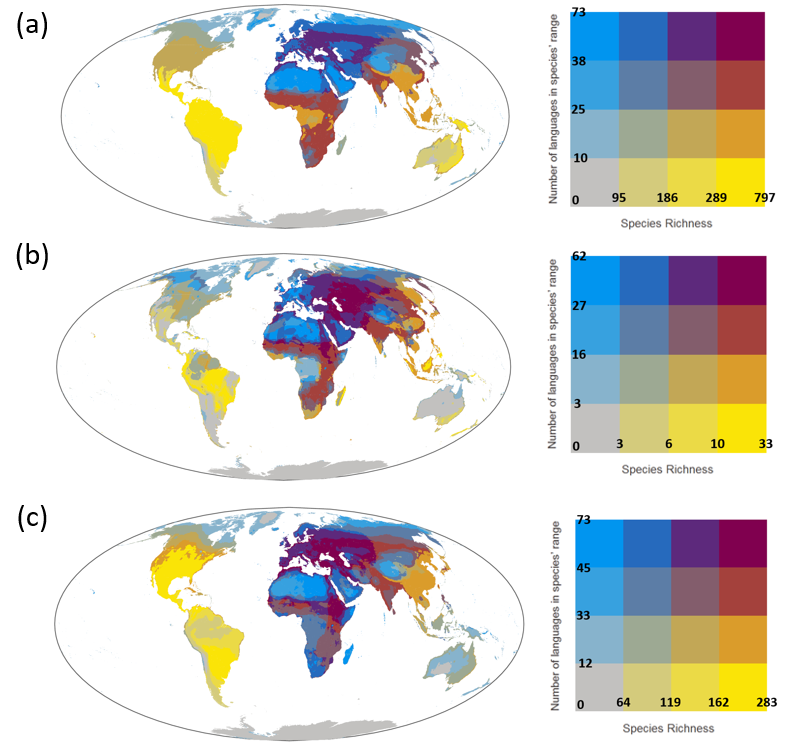


**S5 Figure.** Bivariate maps showing the number of species (species richness) and the mean number of languages within the distribution of species found within each 30km × 30km grid cell for **(a)** all bird species, **(b)** threatened bird species, and **(c)** migratory bird species. The number of languages within each species’ distribution was calculated using the most spoken languages in each country.
